# Mechanism of BIP-4 mediated inhibition of InsP_3_Kinase-A

**DOI:** 10.1042/BSR20211259

**Published:** 2021-07-20

**Authors:** Themistoklis Paraschiakos, Wilhelm Flat, Ya Chen, Johannes Kirchmair, Sabine Windhorst

**Affiliations:** 1 Department of Biochemistry and Signal Transduction, University Medical Center Hamburg-Eppendorf, Martinistrasse 52, D-20246 Hamburg, Germany; 2 Department of Pharmaceutical Sciences, Division of Pharmaceutical Chemistry, University of Vienna, Althanstraße 14, 1090 Vienna, Austria

**Keywords:** InsP3Kinase-A, nitro groups, selective inhibition

## Abstract

Overexpression of the neuronal InsP_3_kinase-A increases malignancy of different tumor types. Since InsP_3_kinase-A highly selectively binds Ins(1,4,5)P_3_, small molecules competing with Ins(1,4,5)P_3_ provide a promising approach for the therapeutic targeting of InsP_3_kinase-A. Based on this consideration, we analyzed the binding mechanism of BIP-4 (2-[3,5-dimethyl-1-(4-nitrophenyl)-1H-pyrazol-4-yl]-5, 8-dinitro-1H-benzo[de]isoquinoline-1,3(2H)-dione), a known competitive small-molecule inhibitor of Ins(1,4,5)P_3_. We tested a total of 80 BIP-4 related compounds in biochemical assays. The results of these experiments revealed that neither the nitrophenyl nor the benzisochinoline group inhibited InsP_3_kinase-A activity. Moreover, none of the BIP-4 related compounds competed for Ins(1,4,5)P_3_, demonstrating the high selectivity of BIP-4. To analyze the inhibition mechanism of BIP-4, mutagenesis experiments were performed. The results of these experiments suggest that the nitro groups attached to the benzisochinoline ring compete for binding of Ins(1,4,5)P_3_ while the nitrophenyl group is associated with amino acids of the ATP-binding pocket. Our results now offer the possibility to optimize BIP-4 to design specific InsP_3_Kinase-A inhibitors suitable for therapeutic targeting of the enzyme.

## Introduction

The inositol-trisphosphate 3-kinase (InsP_3_Kinase) family consists of the three isoenzymes InsP_3_Kinase: A, B, and C. Among these, InsP_3_Kinase-B and C are ubiquitously expressed and mainly control functions of immune cells. InsP_3_Kinase-A, however, is mainly expressed in neurons of the central nervous system (CNS), where it is involved in the control of synaptic plasticity [1]. One interesting characteristic of InsP_3_Kinase-A is its bifunctionality: the enzyme bundles F-actin by its N-terminal actin-binding domain and phosphorylates Ins(1,4,5)P_3_ to Ins(1,3,4,5)P_4_ through its C-terminal catalytic domain [2,3]. Thus, InsP_3_Kinase-A is involved in controlling actin dynamics and Ins(1,4,5)P_3_-mediated calcium signaling.

In addition to neurons of the CNS, different types of tumor cells are able to express InsP_3_Kinase-A by reducing methylation of its promoter, increasing gene body methylation, or mutating the RE1-silencing transcription factor [4,5]. This tumor-specific InsP_3_Kinase-A overexpression has been detected in lung, breast, kidney, skin, and liver cancer. Furthermore, an association of high InsP_3_Kinase-A expression with malignancy of these tumor types was found (reviewed in [3]). Thus, InsP_3_Kinase-A is an interesting target for therapy of tumor diseases. While no compounds exist to inhibit the actin-bundling activity of InsP_3_Kinase-A, several small molecules have been identified to block its InsP_3_Kinase activity [6–9].

The advantage of targeting InsP_3_Kinase-A for therapeutic purposes is its restricted expression in the CNS and its unique catalysis mechanism [3]. In contrast with protein kinases, which utilize only ATP as substrate, InsP_3_Kinase-A binds both ATP and Ins(1,4,5)P_3_. Among the binding pockets, the Ins(1,4,5)P_3_ pocket is unique, while the ATP pocket contains the conserved DFG motif [10]. Thus, targeting the Ins(1,4,5)P_3_ binding loop provides a selective approach to specifically inhibit cellular InsP_3_Kinase-A. In principle, Ins(1,4,5)P_3_ analogs would be powerful and highly selective tools to inhibit cellular InsP_3_Kinase-A activity, but the existing Ins(1,4,5)P_3_ analogs inhibiting InsP_3_Kinase-A activity are not membrane permeable [11,12]. Targeting the highly selective, narrow Ins(1,4,5)P_3_-binding pocket with small molecules is challenging, and this is why in the past mainly ATP competitive InsP_3_Kinase-A inhibitors have been identified [4,7]. However, by performing a high-throughput screen, we were able to identify BIP-4 (2-[3,5-dimethyl-1-(4-nitrophenyl)-1H-pyrazol-4-yl]-5,8-dinitro-1H-benzo[de]isoquinoline-1,3(2H)-dione) as an Ins(1,4,5)P_3_-competitive inhibitor of InsP_3_Kinase-A [9]. BIP-4 inhibited InsP_3_Kinase-A activity with good affinity (IC_50_ = 157 nM) and blocked adhesion and proliferation of lung cancer cells. However, it showed poor membrane permeability [9]. Because of its high affinity towards InsP_3_Kinase-A, BIP-4 is a valuable tool compound to study the molecular mechanism of small molecules binding to the Ins(1,4,5)P_3_ loop. This knowledge should enable the design of membrane-permeable Ins(1,4,5)P_3_-competitive small molecules to selectively block cellular InsP_3_Kinase-A activity. Therefore, in the present study, experiments were performed to investigate the structure-activity relationship of BIP-4 and a large set of analogs to better understand the binding modes of BIP-4 to InsP_3_Kinase-A.

## Materials and methods

### Materials

Ins(1,4,5)P_3_ (potassium salt) was purchased from Buchem B.V. (Apeldoorn, The Netherlands), ATP from ThermoFisher Scientific (Bremen, Germany), and BIP-4 related compounds from ChemDiv (San Diego, California, U.S.A.), ChemBridge Corporation (San Diego, California, U.S.A.) or Vitas M Chemical Limited (Causeway Bay, Hong Kong).

### Bacterial InsP_3_Kinase-A expression and purification

For initial screening, full-length GST-InsP_3_Kinase-A was employed. Therefore, A cDNA fragment encoding the full-length form of InsP_3_Kinase-A was inserted into the vector pGEX-4T-3 encoding an N-terminal glutathione S-transferase (GST) tag. From transformed *Escherichia coli* cell lysates (BL21 DE3), the fusion protein was purified utilizing a glutathione matrix as described [13].

The point mutations Lys209Gln, Lys264Gln, Lys291Gln, Lys312Gln, and Lys419Gln were created by using modified QuickChange® site-directed mutagenesis [14], while point mutations Ser197Ala, Asp262Asn, and Tyr315Phe were mutated using Q5® Site-Directed Mutagenesis Kit (NEB).

For detailed characterization of enzyme kinetics, an eGFP-His-InsP_3_Kinase-A fusion protein was expressed in *E. coli* and after enrichment, eGFP-His was cleaved to produce the native protein in high concentration and quality (see Figure 2A).

For this, the cDNA coding for full-length InsP_3_Kinase-A was adapted for bacterial expression using the IDT Codon Optimization Tool and then cloned into a modified pSF vector (a gift from Aymelt Itzen’s lab, Universitätsklinikum Hamburg Eppendorf), using a sequence- and ligation-independent cloning (SLIC) method [15].

The InsP_3_Kinase-A construct was fused to an N-terminal eGFP, which holds an additional 10× histidine tag. To further purify the construct, a TEV cleavage site was introduced for removing the N-terminal eGFP-His tag. InsP_3_Kinase-A protein was expressed using the *E. coli* C41(DE3) strain in Terrific Broth (TB) medium. With an OD_600_ between 2.0 and 3.0, 0.1 mM isopropyl β-d-1-thiogalactopyranoside (IPTG) was added to induce protein expression. The temperature was changed from 37 to 20°C and maintained overnight for expression in TB medium. The protein was purified using Ni-NTA Agarose (ThermoFisher Scientific, Carlsbad, CA) by following the manufacturer’s protocol. TEV protease was then added to cleave the GFP tag, and proteins were further purified by size exclusion chromatography on a 16/600 Superdex 200 pg column pre-equilibrated with 50 mM Tris, 400 mM NaCl, 3 mM MgCl_2_, and 1 mM Dithiothreitol (DTT).

### ADP Glo assay

The primary screen for InsP_3_Kinase-A inhibitors was performed using the ADP Glo Assay purchased from Promega, containing ADP-Glo reagent, kinase detection reagent, Ultra-Pure ATP, and ADP. It was adapted for use in flat bottom white 96-well plates. The InsP_3_Kinase-A activity was measured indirectly via ATP consumption. As the first step after kinase reaction, ATP is depleted. Next, the newly produced ADP is transformed back to ATP, which serves as a luciferase substrate [16]. In consequence, ATP catalyzed by InsP_3_Kinase-A activity is proportional to the luminescent signal.

For the inhibitor screening, the compounds were diluted in dimethylsulfoxide (DMSO, Sigma), and a final compound concentration of 5 µM was applied to each well. Each measurement was performed in triplicates. As controls, a positive control (enzyme solution with InsP_3_Kinase and without compounds, but with DMSO), a negative control (enzyme buffer without InsP_3_Kinase), and an inhibitor control (quercetin and BIP-4) [7] were applied. In each well, there was a final buffer concentration of 20 mM HEPES pH 7.5, 5 mM MgCl_2_, 30 mM KCl, 1 mM DTT, 0.5 mM ATP, and an enzyme concentration between 20 nM and 55 nM was applied. After incubation for 5 min, Ins(1,4,5)P_3_ with an end-concentration of 100 µM was added, and incubation was continued for 10 min at 30°C. Then, 10 µl of ADP-Glo reagent was added (depleting remaining ATP and stopping InsP_3_Kinase enzymatic reaction) and incubated for 20 min at room temperature (RT). Next, 10 µl kinase detection reagent (converting remaining ADP to ATP and containing luciferase, which uses ATP as substrate) was added, and incubation was continued for 15 min at RT. For luminescent measurement, a TECAN infinite 200 plate reader was used with an integration time of 1000 ms at a temperature of 30°C.

From the screening’s raw data, the mean values of negative controls were used as a reference point with 0%, and the mean values of positive controls were used with 100%, respectively. Those data were analyzed by GraphPad Prism 8. For each plate, the data were grouped, and for each of them, the mean, mean difference between positive control and sample, and standard error of difference were calculated. The most promising hits were validated with the coupled protein kinase/lactate dehydrogenase (PK/LDH) assay.

### Coupled PK/LDH optical assay

The coupled PK/LDH optical assay was used as an orthogonal assay to validate the ADP-Glo Assay data and to determine the inhibition mechanism via Michaelis–Menten dissociation constants (*K*_M_) and the maximum enzyme velocity *V*_Max_. ADP formation is coupled to NADH consumption via pyruvate kinase and lactate dehydrogenase reactions. The assay was adapted for use in flat bottom transparent 96-well plates. The final assay mixture was 0.2 mM NADH, 20 mM HEPES pH 7.5, 5 mM MgCl_2_, 30 mM KCl, 1 mM DTT, 0.5 mM ATP, 1 mM phosphoenolpyruvate, 10 units/ml L-lactate dehydrogenase, and 10 units/ml pyruvate kinase.

For inhibitor validation, the compounds were diluted in DMSO, and a final compound concentration of 5 µM was applied to each well. Each measurement was performed in duplicates. As controls, a positive control (enzyme solution with InsP_3_Kinase and without compounds, but with DMSO), a negative control (enzyme buffer without InsP_3_Kinase), and an inhibitor control (quercetin and BIP-4) [7] were placed. InsP_3_Kinase was added to the mixture to a final concentration of 55 nM. The mixture was further incubated at 30°C for 10 min. After determining the low basal rate of ATP consumption without InsP_3_, maximal enzyme activity was measured by adding InsP_3_ to a final concentration of 30 µM. The concentration of InsP_3_Kinase and Ins(1,4,5)P_3_ was adjusted accordingly in the beginning so that the activity persisted for min at *V*_Max._ The final volume of the assay was 300 µl per well. The absorption data was collected using a TECAN infinite 200 plate reader, and the enzyme activity was calculated from the following equation:

$$enzyme\;activity\;\left[\frac{M}{min}\right]=-\frac{dA_{365}}{dt}\left[\frac{1}{min}\right]\times \frac{1}{\varepsilon }\times \frac{1}{l}$$

$$\varepsilon \;\times \;l=\;2899.1\;\left[\frac{1}{M}\right]$$

ε = molar absorbance coefficient, *l* = optical path length

The rates are then corrected for background NADH decomposition of controls containing no InsP_3_Kinase and transformed into a fitting unit. From these data, the mean values of negative controls were used as a reference point with 0%, and the mean values of positive controls were used with 100%, respectively.

Since the *K*_M_ value for Ins(1,4,5)P_3_ is very low [7], inhibitor type with respect to Ins(1,4,5)P_3_ was analyzed in a spectrophotometer, employing different Ins(1,4,5)P_3_ concentrations, starting with 2.4 µM, in a final volume of 500 µM. Enzyme activity was calculated as follows:

$$enzyme\;activity\left[\frac{M}{min}\right]=-\frac{dA_{365}}{dt}\left[\frac{1}{min}\right]\times \frac{1}{\varepsilon }\times \frac{1}{l}$$

$$\varepsilon \times l=3400\left[\frac{1}{M}\right]$$

### Testing inhibition of the indicator enzymes

To identify compounds inhibiting the indicator enzymes of the ADP Glo or the coupled optical assay, the assays were performed in the absence of InsP_3_Kinase. Therefore, the standard reaction mix was incubated at 30°C for 10 min in the presence and absence of inhibitors without adding InsP_3_Kinase. About 100 µM ADP was used to start the reaction, and the observed rapid consumption of NADH was recorded, and the enzyme activity of the coupled enzymes was calculated.

### Determination of *K*_M_ and *V*_Max_

*K*_M_ for ATP was analyzed at varying ATP concentrations of 10, 25, 50, 100, 200, and 500 µM and a constant Ins(1,4,5)P_3_ concentration of 30 µM. *K*_M_ for Ins(1,4,5)P_3_ was analyzed at 2.4, 6, 12, 18 µM Ins(1,4,5)P_3_ and at constant ATP concentration (500 µM). For each measurement, an inhibitor concentration of 20 µM was applied. *K*_M_ and *V*_Max_ were evaluated by GraphPad Prism 8, as well as by Lineweaver–Burk Plot, and an inhibition mechanism was determined.

### Thermal shift assay

To monitor protein unfolding, the fluorescent Protein Thermal Shift™ Dye Kit was used. The unfolding process exposes the hydrophobic region of proteins and results in a significant increase in fluorescence, which monitors the protein-unfolding transition. The thermal shift assay was conducted in the QuantStudio 3 Real-Time-PCR-System (ThermoFisher Scientific, Carlsbad, CA, U.S.A.), initially designed for PCR. The system contains a heating/cooling device for accurate temperature control and a charge-coupled device (CCD) detector for simultaneous imaging of the fluorescence changes in the microplate wells. The final concentration of InsP_3_Kinase-A wildtype (wt) and mutants was 6.7 µM, and the final compound concentration was 20 μM. The plate was heated from 25 to 99°C with a heating rate of 0.05°C/s. The fluorescence intensity was measured with *E*x/*E*m: 580/623 nm. The fluorescence imaging data from the CCD detector were analyzed using the Protein Thermal Shift™ Software v1.4 (ThermoFisher Scientific, Carlsbad, CA), and derivative Tm values were obtained.

### Docking studies

3D structure of BIP-4 was obtained from MolPort (Riga, Latvia). The crystal structure of InsP_3_Kinase-A (PDB code: 1W2C) was obtained from the Protein Data Bank. The ligands within the crystal structure complex were extracted by PyMOL software (San Carlos, CA, U.S.A.). AutoDock 4.2 was used for the docking system test. AutoDock tools initialized the ligands by adding gasteiger charges, merging nonpolar hydrogen bonds, and setting rotatable bonds. The ligands were rewritten into PDBQT format, which can be read by Autodock software (AutoDock 4.2, San Carlos, CA, U.S.A.). AutoDock Tools were used to add polar hydrogen to the entire receptor. The grid box was set to contain the entire active center region. The receptor output was also saved in PDBQT format. AutoDock was set with the macromolecule held fixed and the ligands flexible. Affinity maps for all the atom types present, as well as an electrostatic map, were computed, with a grid spacing of 0.375 Å. The structural models were collected from the lowest-energy docking solution of each cluster of autodocks and visualized with PyMOL software.

## Results and discussion

### Inhibition of InsP_3_Kinase-A activity by molecular fragments and analogs of BIP-4

Previous work identified two plausible orientations of BIP-4 in which the small molecule could bind to the Ins(1,4,5)P_3_ binding pocket of InsP_3_Kinase-A (Supplementary Figure S1 and [9]). The first plausible binding mode is characterized by the alignment (hence, competition) of the two nitro groups attached to the benzisochinoline group with the phosphate moieties in positions 1 and 4 of Ins(1,4,5)P_3_ (P1 and P4). The second potential binding mode is characterized by the overlap between the nitro group attached to the terminal phenyl ring of BIP-4 and the phosphate moiety in position 1 of Ins(1,4,5)P_3_ [9].

In order to understand whether any of the three nitro moieties of BIP-4 is essential to its inhibitory activity on InsP_3_kinase-A, we tested three commercially available fragments of BIP-4 (**1, 2,** and **3**) in an ADP-Glo™ Kinase Assay, with quercetin [8] and BIP-4 [13] serving as control (Table 1). These three compounds essentially represent substructures of BIP-4: **1** and **2** lack the nitrophenyl and pyrazole moieties whereas **3** lacks the benzisochinoline ligand core. None of the three substances showed any significant bioactivity, indicating that one or several of the nitro groups of BIP-4 are essential for bioactivity.

**Table 1:** Structures of molecular fragments of BIP-4

Compound number	1	2	3
**ID (Supplier)**	8004-3428 (ChemDiv Inc.)	8004-3436 (ChemDiv Inc.)	2425-2506 (ChemDiv Inc.)
**Structural name**	2-methyl-5, 8-dinitro- benzo[de]isoquinoline-1, 3-dione	2-ethyl-5, 8-dinitro- benzo[de]isoquinoline-1, 3-dione	3,5-dimethyl-1-(4-nitro-phenyl)- 1H-pyrazole
**Chemical structure**	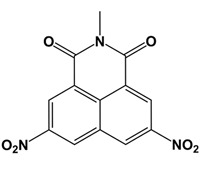	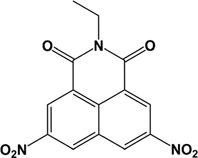	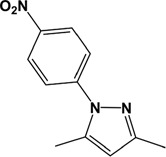
**ADP Glo Assay**			
**Activity difference in % at 5 µM (SD)**	-6.52 (4.99) *P*=1	14.87 (2.17) *P*=0.999	0.90 (6.98) *P*=1

Compounds **1** and **2** are benzisochinoline fragments, and compound **3** represents the nitrophenol and the pyrazole moiety.

Along with the three fragments we purchased and tested 77 further derivatives of BIP-4 to improve our understanding of the structure–activity relationship and the binding of BIP-4 to InsP_3_Kinase-A. Based on molecular scaffolds, 71 of the 77 compounds can be assigned to three different groups of compounds (Supplementary Tables S1–4): The first group is defined by a molecular scaffold that is most closely related to BIP-4 among all the structures, with a pyrazole moiety substituted at the N of the benzisochinoline ligand core. With the exception of compound **13**, all compounds assigned to this group carry at least one nitro moiety (Supplementary Table S1). In the second group of compounds, the pyrazole moiety is replaced by a benzene. Most compounds of this group have two nitro substituents attached to the benzisochinoline ligand core, while substitutions of the benzene moiety vary (Supplementary Table S2). The third group of compounds includes a linker between the benzisochinoline and the benzene moieties, with different types and lengths of linkers (Supplementary Table S3). Six of the 77 compounds could not be assigned to any of the three groups; they are singletons (Supplementary Table S4).

For this screen, all measurements were performed in triplicates, three-times using the ADP Glo assay. After the first calculation of mean values, those compounds exhibiting the highest inhibitory activity were re-evaluated (*n*=4–8, see Supplementary Table S5). That is why for instance compound **54** (*n*=3) has no significant inhibitory activity (*P*=0.06) but compound **55** (*n*=6) has (*P*=0.04).

**Figure 1:**
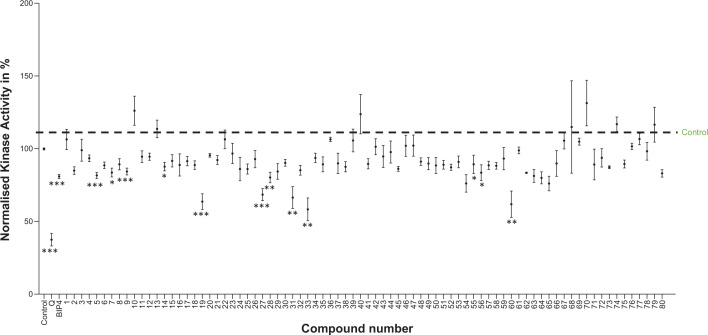
Effect of BIP-4 related compounds on InsP_3_Kinase-A activity Full length GST-InsP_3_Kinase activity was measured by the ADP-Glo™ Kinase Assay in the absence of small molecules (control) or in the presence of 1 µM quercetin (Q), 5 µM BIP-4, or 5 µM of 77 BIP-4 related compounds; *P<0.05, **P<0.005, ***P<0.0001.

In group one, we found four significant active compounds (**5, 7, 9, 14**), in group two seven (**19, 27, 28, 31, 33, 55, 56**), and in group three one (**60**) (Supplementary Table S5 and Figure 1). Among these, we re-evaluated compounds **5, 7, 9, 19, 27, 31, 33, 60** (Table 2) for inhibition of indicator enzymes as well as by the coupled PK/LDH optical assay.

**Figure 2:**
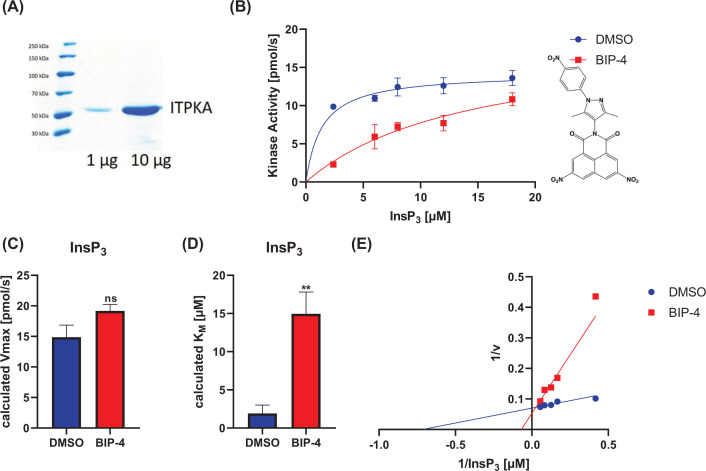
Purification of full-length InsP_3_Kinase-A and validation of BIP-4 (**A**) Full-length InsP_3_Kinase-A was expressed in *E. coli* as an eGFP-His-fusion protein and purified by Ni-NTA Agarose. The eGFP-His-tag was cleaved and the protein purified by size exclusion chromatography. Purity and concentration of InsP_3_Kinase-A were analyzed by SDS-PAGE. This tag-less protein was used for all further assays (Figures 3, 4 and 6). (**B**) The effect of BIP-4 on InsP_3_Kinase-A activity was measured at different Ins(1,4,5)P_3_ concentrations. (**C, D**) The experiment was performed three-times, and *V*_max_ and *K*_M_ were calculated. **P<0.005. **(E)** A Lineweaver–Burk plot was performed to determine the type of inhibition.

**Table 2:** Chemical structure and names of active compounds

Compound number	5	7	9	19
(A)				
**ID (Supplier)**	7045306 (ChemBridge Corp.)	7093221 (ChemBridge Corp.)	7053478 (ChemBridge Corp.)	8003-3839 (ChemDiv Inc.)
**Structural name**	3-(3,5-dimethyl-1H-pyrazol-4-yl)-7-nitro-3-azatricyclo[7.3.1.0^5,13^] trideca-1(13),5,7,9,11-pentaene- 2,4-dione	3-(1-ethyl-3,5-dimethyl-1H-pyrazol-4-yl)-7-nitro-3-azatricyclo[7.3.1.0^5,13^]trideca-1(13),5,7,9,11-pentaene- 2, 4-dione	7-bromo-3-[3,5-dimethyl-1-(4-nitrophenyl)-1H-pyrazol-4-yl]-3-azatricyclo[7.3.1.0^5,13^]trideca-1(13),5,7,9,11-pentaene- 2, 4-dione	2-(2,4-dimethyl-phenyl)-5,8-dinitro-benzo[de]isoquinoline- 1,3-dione
**Chemical structure**	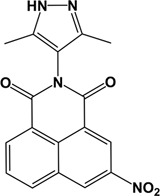	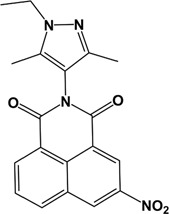	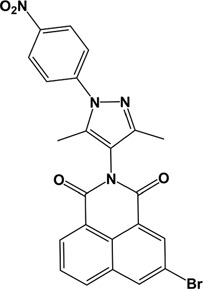	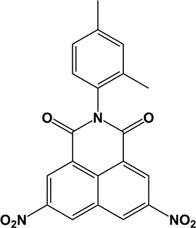
**ADP Glo Assay**				
**Activity difference in % at 5 µM (SD)**	18.21 (1.81) *P*<0.0001	16.22 (3.12) *P*=0.031	15.53 (2.20) *P*<0.0001	36.22 (4.04) *P*<0.0001
**Assay Inhibitor**	No	No	No	Yes
**PK/LDH Assay**				
**Activity difference in % at 5 µM (SD)**	11.26 (0.1141)	4.266 (0.03669)	10.59 (0.07587)	-
**Assay Inhibitor**	No	No	No	Yes

Compound number	27	31	33	60
(B)				
**ID (Supplier)**	8003-3844 (ChemDiv Inc.)	8003-3811 (ChemDiv Inc.)	8004-6146 (ChemDiv Inc.)	8004-3379 (ChemDiv Inc.)
**Structural name**	2-(4-ethoxy-phenyl)-5,8-dinitro-benzo[de]isoquinoline-1,3-dione	4-(5,8-dinitro-1,3-dioxo-1H,3H-benzo[de]isoquinolin-2-yl)-N-methyl-benzenesulfonamide	4-(5,8-dinitro-1,3-dioxo-1H,3H-benzo[de]isoquinolin-2-yl)-N,N-diethyl-benzenesulfonamide	6,7-dinitro-2-o-tolylamino-benzo[de]isoquinoline-1,3-dione
**Chemical structure**	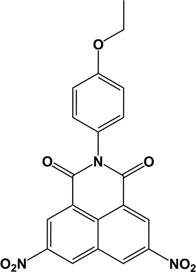	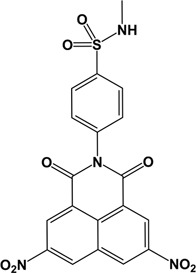	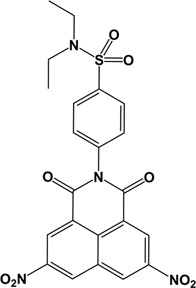	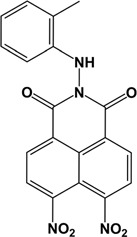
**ADP Glo Assay**				
**Activity difference in % at 5 µM (SD)**	31.43 (3.34) *P*<0.0001	33.41 (5.53) *P*=0.0015	41.56 (5.92) *P*=0.0003	38.02 (6.86) *P*=0.035
**Assay Inhibitor**	Yes	Yes	Yes	Yes
**PK/LDH Assay**				
**Activity difference in % at 5 µM (SD)**	-	-	-	-
**Assay Inhibitor**	Yes	Yes	Yes	Yes

Thereby, we found that all compounds of groups two and three (**19, 27, 31, 33, 60**) also inhibited indicator enzymes (Table 3A,B), indicating that they likely are false-positive hits. Only three compounds inhibited InsP_3_Kinase-A and not luciferase: compounds; **5, 7,** and **9** (Table 3A). The activities of these compounds were in a similar range to that of BIP-4 (Figure 1 and Table S5). In conclusion, from the BIP-4 related compounds, only closely related compounds were real hits (Supplementary Table S1), while the apparent hits of groups 2 and 3 were false positive.

**Table 3:** Site-directed mutagenesis of InsP_3_Kinase-A

Mutant	Enzyme activity in %	Enzyme activity + BIP4 in %
**WT**	100 ± 16.53	69.48 ± 18.09
**Ser197Ala**	43.80 ± 21.84	47.76 ± 28.17
**Lys209Gln**	0	not tested
**Asp262Asn**	14.91 ± 18.97	40.59 ± 21.32
**Lys264Gln**	45.66 ± 12.84	18.797 ± 5.29
**Lys291Gln**	0	not tested
**Lys312Gln**	0	not tested
**Tyr315Phe**	9.89 ± 7.23	56.81 ± 10.44
**Lys419Gln**	46.12 ± 8.78	56.96 ± 7.83

Different InsP_3_Kinase-A mutants were created by QuickChange® site-directed mutagenesis [14] and Q5® Site-Directed Mutagenesis Kit (NEB). The respective proteins were expressed in *E. coli*, purified, and InsP_3_Kinase-A activity was measured in absence (neg) or in presence of BIP-4. Shown are mean values ± SD of three independent measurements.

### ATP and Ins(1,4,5)P_3_ -competition studies of BIP-4 analogs

Before the BIP-4 related compounds were characterized, we evaluated that the full-length form of InsP_3_Kinase-A, employed in this study, acts competitive to Ins(1,4,5)P_3_ using the coupled PK/LDH optical assay. For this purpose, both assays conditions and protein quality were optimized (see methods and Figure 2A). These conditions were employed for all further experiments.

Calculation of *V*_Max_ and *K*_M_ values (Figure 2B–D), as well as the Lineweaver–Burk Plot (Figure 2E), revealed that BIP-4 competed for binding of Ins(1,4,5)P_3._ In addition, we calculated a *K*_I_ value of 437 nM (Supplementary Figure S2). However, the IC_50_ value of BIP-4 for the catalytic domain of InsP_3_Kinase-A is 157 nM [9]. Thus, the affinity of BIP-4 to the full-length protein is lower compared with the catalytic domain. We assume that the N-terminal InsP_3_Kinase-A domain impedes binding of BIP-4 to the Ins(1,4,5)P_3_ binding pocket.

**Figure 3:**
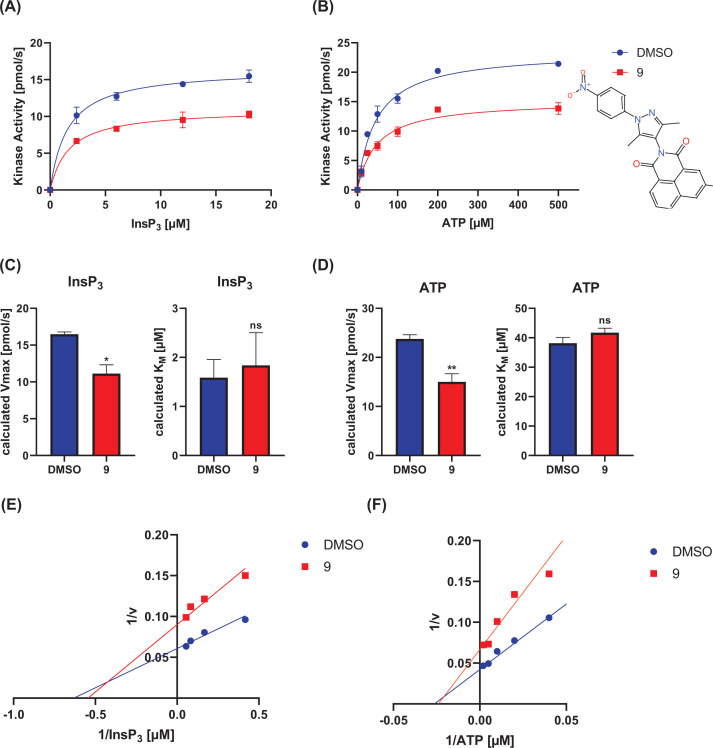
Inhibitor type of compound 9 *K*_M_ and *V*_Max_ with respect to ATP and Ins(1,4,5)P_3_ were determined in presence of 20 µM compound **9**. (**A** and **B**) Representative measurements. (**C** and **D**) The experiment was performed three-times, and *V*_max_ and *K*_M_ were calculated (*P<0.05, **P<0.005). (**E** and **F**) Lineweaver–Burk plots were performed to determine the type of inhibition.

**Figure 4:**
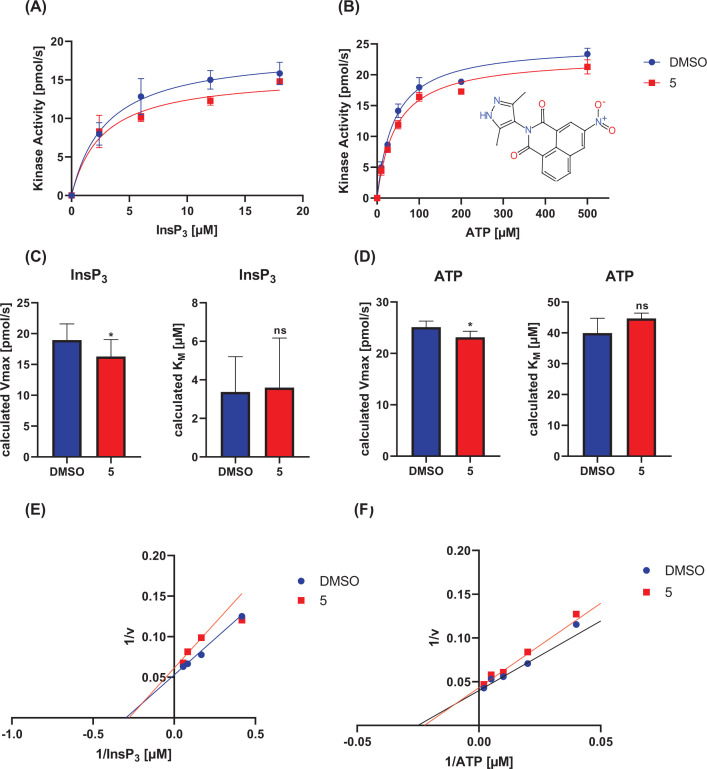
Inhibitor type of compound 5 *K*_M_ and *V*_Max_ with respect to ATP and Ins(1,4,5)P_3_ were determined in presence of 20 µM compound **5.** (**A** and **B**) Representative measurements. (**C** and **D**) The experiment was performed three-times, and *V*_max_ and *K*_M_ were calculated (*P<0.05). (**E** and **F**) Lineweaver–Burk plots were performed to determine the type of inhibition.

To analyze whether BIP-4 related compounds also act competitively to Ins(1,4,5)P_3_, the two compounds of group one showing the highest significance (**5** and **9**) were tested under the same experimental conditions as BIP-4. For this, different inhibitor (10, 20, 40 µM) and substrate concentrations (see Materials and methods section) were employed (Supplementary Figures S3 and S4). For statistical evaluation, *V*_Max_ and *K*_M_ values were determined again at 20 µM inhibitor concentration (*n*=3), and the mean values (Figures 3 and 4A–D), as well as Lineweaver–Burk plots (Figures 3 and 4E,F), were calculated to determine the inhibitor type. This analysis revealed that for both compounds and substrates, *V*_Max_ significantly decreased, but *K*_M_ was not changed. This result, as well as the Lineweaver–Burk Plot, indicate that compounds **5** and **9** are not competitive to ATP and to Ins(1,4,5)P_3_, thus belong to the group of allosteric inhibitors. This result shows that the BIP-4-related compounds exhibit a different inhibitor mechanism compared with BIP-4, emphasizing the unique property of BIP-4.

In conclusion, none of the tested BIP-4 analogs was observed to compete with Ins(1,4,5)P_3_ for binding.

### Analysis of InsP_3_Kinase-A mutants to predict the binding of BIP-4 inside the catalytic domain

**Figure 5:**
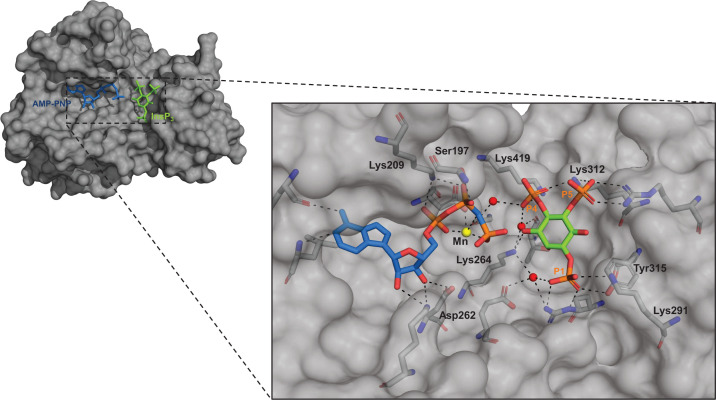
Structure of InsP_3_Kinase-A kinase domain (surface representation in grey) in presence of the substrate analog AMP-PNP (shown as blue sticks) and Ins(1,4,5)P_3_ (shown as green sticks) [10] The zoom shows Ins(1,4,5)P_3_ and AMP-PNP with their interacting polar amino acids. The color code is as follows: orange phosphates and red oxygen. Water is represented as red spheres.

The fact that BIP-4 exhibits such specific activity prompted us to investigate its mechanism of action with biochemical methods. For this purpose, BIP-4 was docked inside the Ins(1,4,5)P_3_ pocket (see Materials and methods), and after trying different configurations, the following mutants were created: Ser197Ala, Lys209Gln, Asp262Asn, Lys264Gln, Lys291Gln, Lys312Gln, Tyr315Phe, Lys419Gln (Figure 5). Among these, Lys209Gln, Lys291Gln, and Lys312Gln were inactive, while the other mutants showed 10–55% enzyme activity compared with wt InsP_3_Kinase-A (Table 3). Therefore, Ser197Ala, Asp262Asn, Lys264, Tyr315Phe, and Lys419Gln were tested for BIP-4 inhibition. The result of these experiments showed that BIP-4 did not inhibit enzyme activity of mutants Ser197Ala and Lys419Gln (Table 3). Interestingly, enzyme activities of Asp262Asn and Tyr315Phe were even increased in presence of BIP-4 (Table 3). Since these mutants exhibit a strongly reduced enzyme activity compared with wt enzyme (85% and 90%, respectively), we assumed that folding of the proteins might be altered. To analyze this assumption, a thermal shift assay was performed. This assay measures the temperature required to denaturate an enzyme. In its unfolded conformation, hydrophobic amino acids are exposed where the dye can bind to and generates a signal. A ligand typically stabilizes a protein, and a mutation can result in stabilization or destabilization [17].

**Figure 6:**
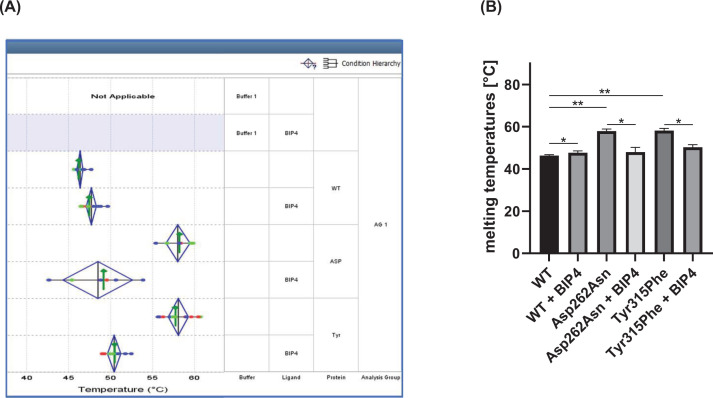
Effect of InsP_3_Kinase-A mutations and BIP-4 binding on protein folding (**A**) A thermal shift assay was performed to compare stability of wt InsP_3_Kinase-A and mutants in presence and absence of BIP-4. (**B**) The assay was performed three times, and the mean temperature ± SD required to unfold the protein was calculated and depicted as bar graph; *P<0.05, **P<0.005.

The result of this assay revealed that BIP-4 increased the temperature by 1.34°C when bound to wt enzyme, confirming binding of BIP-4 to InsP_3_Kinase-A. Mutations of Asp262 to Asn or Tyr315 to Phe stabilized the protein even to a higher extend than BIP-4 (Δ*T* increase 9.97°C and 7.83°C). Interestingly, in presence of BIP-4, the temperature necessary to denaturate the mutant proteins was similar to that of wt InsP_3_Kinase-A (Figure 6A,B). From this result, we conclude that mutations of Asp262 to Asn and Tyr315 to Phe narrow the substrate-binding pocket, impeding binding and/or turnover of substrates. Binding of BIP-4 seems to open the binding pockets and thereby facilitates catalysis. However, this result also shows that Asp262 and Tyr315 are not essential to bind to BIP-4.

**Figure 7:**
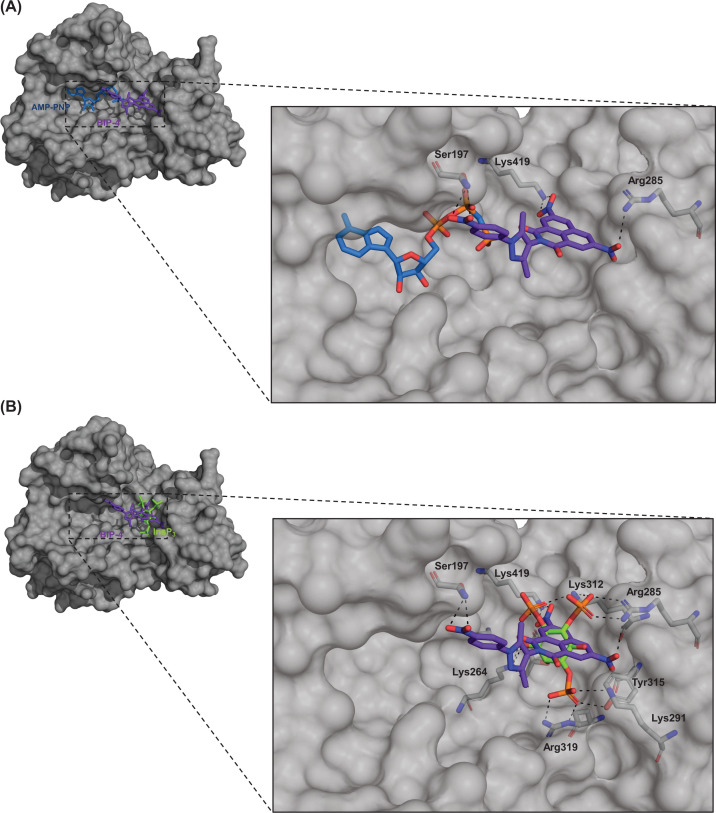
Docking of BIP-4 into the catalytic domain of InsP_3_Kinase-A A detailed description of docking parameters is given in methods. Green: InsP(1,4,5)P_3_, blue: ATP, purple: BIP-4. (**A**) BIP-4 in presence of ATP. (**B**) BIP-4 in presence of InsP(1,4,5)P_3_.

In conclusion, our mutant studies revealed that Ser197 and Lys419 are essential for BIP-4 binding. This result is very interesting because it indicates that the ATP binding pocket is involved in binding of BIP-4. Indeed, a random docking study also suggested the involvement of the ATP binding pocket into BIP-4 binding. In Figure 7A, a 3-D model of the catalytic domain of InsP_3_Kinase-A in presence of ATP and BIP-4, and in Figure 7B in presence of Ins(1,4,5)P_3_ and BIP-4 is shown. Here, the nitrophenyl group extends into the ATP pocket and interacts with amino acids involved in binding of ATP phosphate groups. The pyrazole moiety serves as linker, and the benzisochinoline ring binds into the Ins(1,4,5)P_3_ binding pocket and thereby competes for binding of Ins(1,4,5)P_3_. This model is only partly in line with our previous prediction, suggesting that the nitrophenyl-group was positioned opposite to the ATP binding pocket (Supplementary Figure S1). In addition, the newly suggested model explains why compounds **5** and **9,** lacking the nitrophenyl ring or the nitro groups at the benzisochinoline group, are insufficient to compete for binding of Ins(1,4,5)P_3_ and why the single fragments of BIP-4 are not bioactive.

## Conclusions

In the last past years, increasing evidence has been presented that overexpression of InsP_3_Kinase-A increases malignancy of different types of tumor cells [3,18,19]. However, therapeutic approaches to selectively block the malignant potential of InsP_3_Kinase-A have not been developed yet. In the present study, we validated the high selectivity of BIP-4 and revealed that all molecular fragments are essential to inhibit enzyme activity of InsP_3_Kinase-A. BIP-4 could therefore serve as a valuable starting point for the development of new, effective antitumor agents, exhibiting improved membrane permeability.

## Supplementary Material

Supplementary Figures S1-S4 and Tables S1-S5

## Data Availability

All supporting data are included within the main article and its supplementary files. Links to database are given in Methods.

## Abbreviations

ADP: adenosine diphosphate
ATP: adenosine triphosphate
BIP-4: 2-[3,5-dimethyl-1-(4-nitrophenyl)-1H-pyrazol-4-yl]-5,8-dinitro-1H-benzo[de]isoquinoline-1,3(2H)-dione
cDNA: complementary DNA
CNS: central nervous system
DMSO: dimethylsulfoxide
DTT: dithiothreitol
*E. coli*: *Escherichia coli*
HEPES: 4-(2-hydroxyethyl)-1-piperazineethanesulfonic acid)
Ins(1,3,4,5)P_4_: inositol 1,3,4,5*-*tetrakisphosphate
Ins(1,4,5)P_3_: inositol 1,4,5-trisphosphate
InsP_3_kinase-A: inositol 1,4,5-trisphosphate-kinase-A
KCl: potassium chloride
MgCl_2_: magnesiumchloride
NADH: nicotinamide adenine dinucleotide
P: phosphate
