# Supplementary material for: Mechanism of BIP-4 mediated inhibition of InsP3Kinase-A
Source: Biosci Rep. 2021 Jul 20;41(7):BSR20211259. doi: 10.1042/BSR20211259 (PMC8292763; doi:10.1042/BSR20211259)
Supplement: Supplementary Figures S1-S4 and Tables S1-S5 [file BSR-2021-1259_supp.pdf]

Figure S1

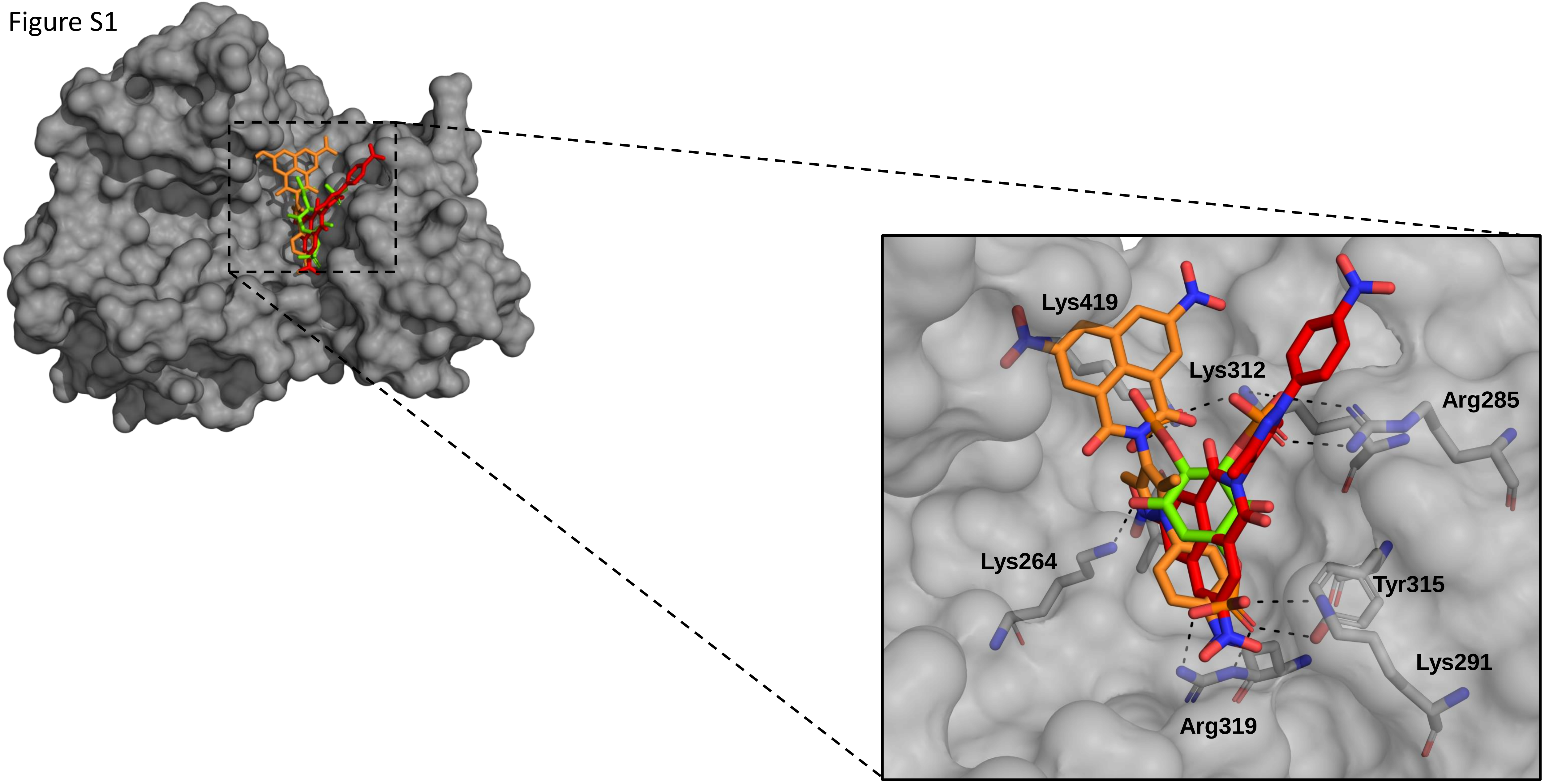

- **Fig. S1. Docking of BIP-4 into the substrate binding pocket of $InsP_3$Kinase-A, published in Schröder et al. 2015 [9].**

Figure S2

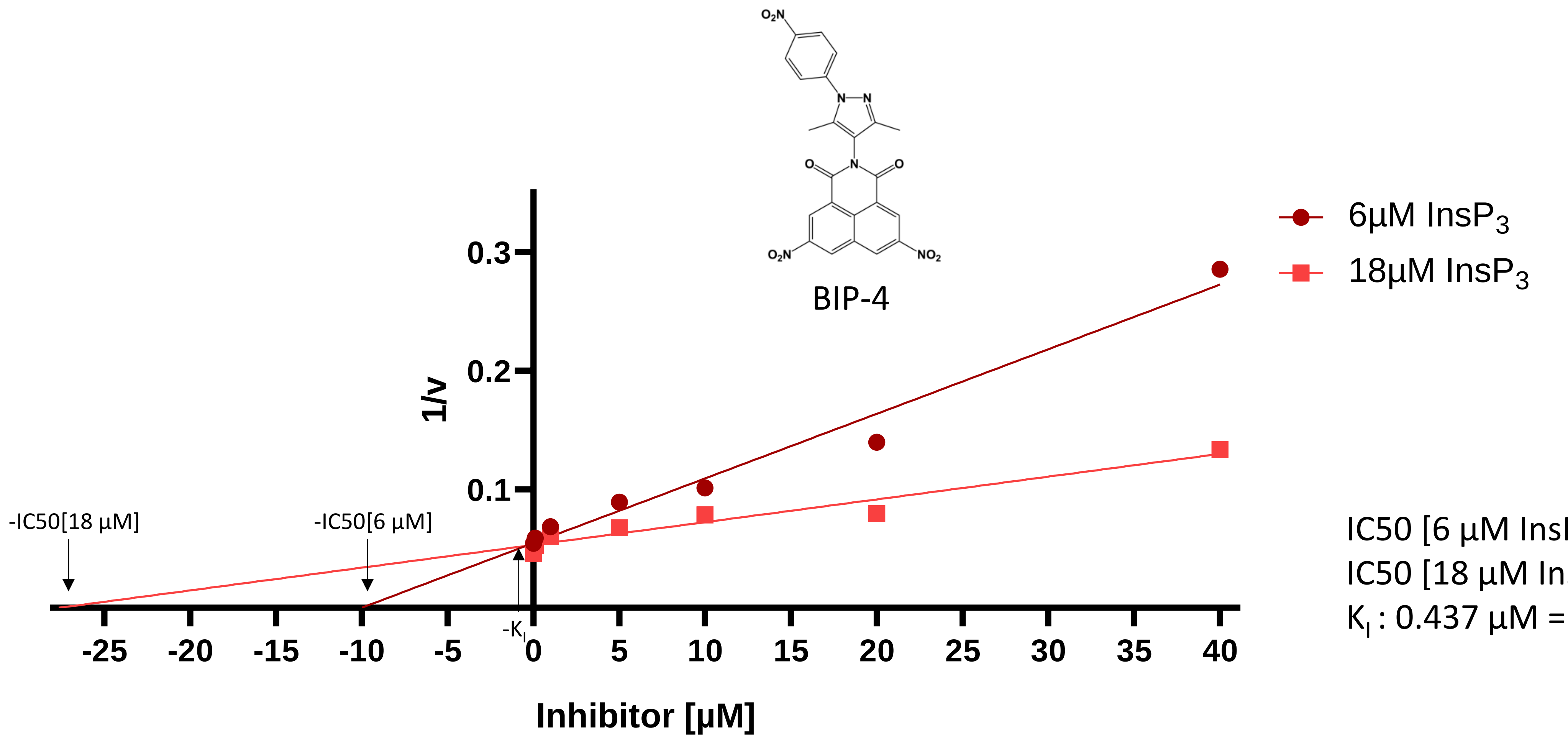

IC50 [6 µM $InsP_3$]: 10.03 µM BIP4
IC50 [18 µM $InsP_3$]: 27.67 µM BIP4
K_I : 0.437 µM = 437 nM

- **Fig. S2. Determination of K_I value for BIP-4.** This assay was performed under optimized conditions using the coupled optical assay, described in methods.

Figure S3

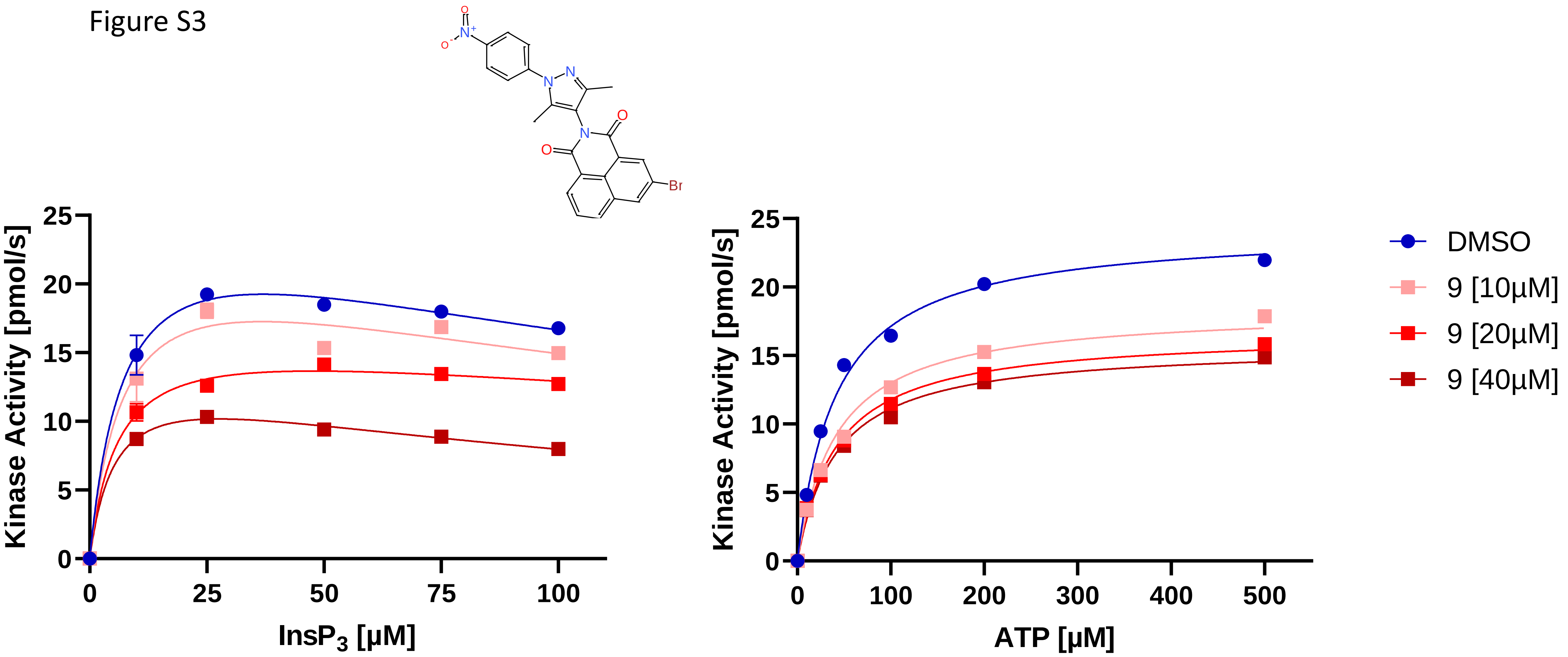
O
N+
O-
N
N
O
N
O
Br
Kinase Activity [pmol/s]
25
20
15
10
5
0
0
25
50
75
100
InsP3 [μM]
Kinase Activity [pmol/s]
25
20
15
10
5
0
0
100
200
300
400
500
ATP [μM]
DMSO
9 [10μM]
9 [20μM]
9 [40μM]

- **Fig. S3 Measurement of $InsP_3$Kinase-A activity at different substrate and compound 9 concentrations. T**his assay was performed under optimized conditions using the coupled optical assay, described in methods.

Figure S4

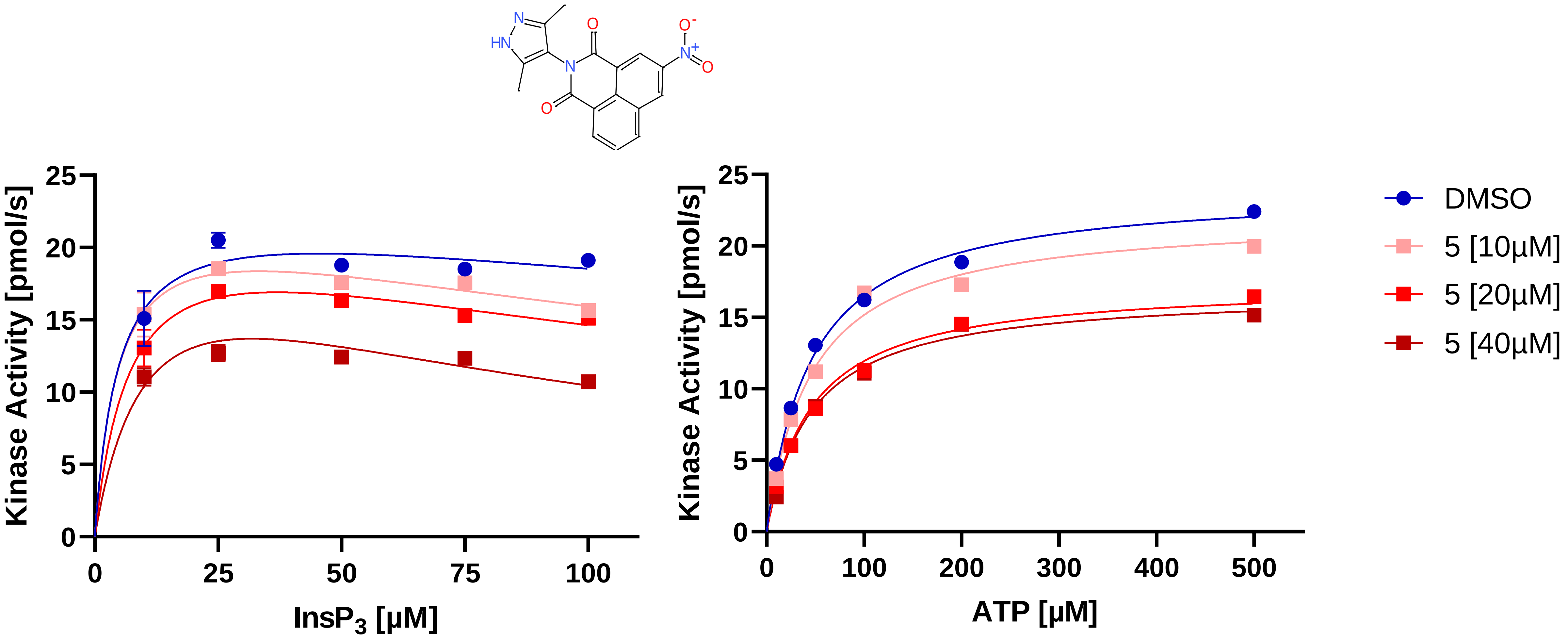
HN
N
N
O
O
O-
N+
O
Kinase Activity [pmol/s]
25
20
15
10
5
0
0
25
50
75
100
InsP3 [µM]
Kinase Activity [pmol/s]
25
20
15
10
5
0
0
100
200
300
400
500
ATP [µM]
DMSO
5 [10µM]
5 [20µM]
5 [40µM]

- **Fig. S4 Measurement of $InsP_3$Kinase-A activity at different substrate and compound 5 concentrations. T**his assay was performed under optimized conditions using the coupled optical assay, described in methods.

Table S1-4. Chemical classes of BIP-4 analogs.

Table S1. BIP-4 analogs (group 1).

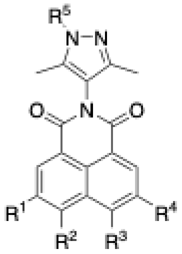

Compound number	Supplier	Supplier ID	R^1	R^2	R^3	R^4	R^5	% inhibition[1]	SD	P value
**BIP-4**	ChemDiv Inc.	8010-1372	$-NO_2$	-	-	$-NO_2$	$-4-PhNO_2$	18.94	1.48	<0.0001
**4**	Vitas-M	STK761945	$-NO_2$	-	-	$-NO_2$	-H	6.44	2.07	0.3470
**5**	ChemBridge Corp.	7045306	-	-	-	$-NO_2$	-H	18.21	1.81	<0.0001
**6**	ChemDiv Inc.	8010-2222	-	-	-	$-NO_2$	$-CH_3$	11.27	2.35	0.0501
**7**	ChemBridge Corp.	7093221	-	-	-	$-NO_2$	$-C_2H_5$	16.22	3.12	0.0313
**8**	ChemBridge Corp.	7034145	-	-	-	$-NO_2$	-Ph	10.58	3.36	0.3330
**9**	ChemBridge Corp.	7053478	-	-	-	-Br	$-4-PhNO_2$	15.53	2.20	<0.0001
**10**	Vitas-M	STK371761	-	$-NO_2$	-	-	$-CH_3$	-26.29	7.64	0.2416
**11**	Vitas-M	STL393078	-	$-NO_2$	-	-	-Ph	5.32	3.67	0.9875
**12**	ChemDiv Inc.	8010-2221	-	-Br	-	-	$-4-PhNO_2$	5.31	2.50	0.8195
**13**	Vitas-M	STK374881	-	-	$-NH_2$	-	$-CH_3$	-13.74	4.70	0.4270
**14**	ChemDiv Inc.	8010-2470	-	-	-	-	$-4-PhNO_2$	12.13	2.33	0.0310

[1] [1] With 5µM Compound, measured with ADP Glo™ Assay, normalized to positive control

Table S2. BIP-4 analogs (group 2).

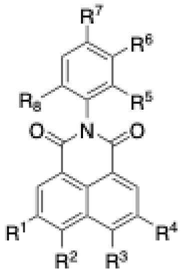

Compound number	Supplier	Supplier ID	R^1	R^2	R^3	R^4	R^5	R^6	R^7	R^8	% inhibition[1]	SD	P value
**15**	ChemDiv Inc.	8004-3462	$-NO_2$	-	-	$-NO_2$	-	-	$-NO_2$	-	8.17	5.23	0.9346
**16**	ChemDiv Inc.	8004-3456	$-NO_2$	-	-	$-NO_2$	-	$-NO_2$	-	-	10.98	4.15	0.6285
**17**	ChemDiv Inc.	8004-3411	$-NO_2$	-	-	$-NO_2$	-	$-CH_3$	-	-	8.41	4.34	0.8318
**18**	ChemDiv Inc.	8003-3829	$-NO_2$	-	-	$-NO_2$	-	$-CH_3$	$-CH_3$	-	11.11	3.64	0.4236
**19**	ChemDiv Inc.	8003-3839	$-NO_2$	-	-	$-NO_2$	$-CH_3$	-	$-CH_3$	-	36.22	4.04	<0.0001
**20**	ChemDiv Inc.	8004-3305	$-NO_2$	-	-	$-NO_2$	-	$-CH_3$	-	$-CH_3$	4.42	2.38	0.8549
**21**	ChemDiv Inc.	8004-3445	$-NO_2$	-	-	$-NO_2$	-	-	-COOH	-	7.66	2.84	0.5576
**22**	ChemDiv Inc.	8003-3780	$-NO_2$	-	-	$-NO_2$	-	-COOH	-	-	-6.58	4.82	0.9938
**23**	ChemDiv Inc.	8004-3440	$-NO_2$	-	-	$-NO_2$	-COOH	-	-	-	3.15	5.87	0.9995
**24**	ChemDiv Inc.	8004-3420	$-NO_2$	-	-	$-NO_2$	-	-	$-NHCOCH_3$	-	13.82	5.04	0.5376
**25**	ChemDiv Inc.	8004-3318	$-NO_2$	-	-	$-NO_2$	-	-	-OH	-	13.78	1.25	0.0576
**26**	ChemDiv Inc.	8010-2482	$-NO_2$	-	-	$-NO_2$	$-OCH_3$	-	-	-	7.01	4.05	0.9346

**27**	ChemDiv Inc.	8003-3844	-NO_2	-	-	-NO_2	-	-	-OC_2H_5	-	31.43	3.34	<0.0001
**28**	ChemDiv Inc.	8004-3310	-NO_2	-	-	-NO_2	-Cl	-	-	-	19.66	3.34	0.0006
**29**	ChemDiv Inc.	8003-3836	-NO_2	-	-	-NO_2	-	-	-Br	-	15.52	2.28	0.1444
**30**	ChemDiv Inc.	8003-3841	-NO_2	-	-	-NO_2	Ph	-	-	-	9.55	3.10	0.5222
**31**	ChemDiv Inc.	8003-3811	-NO_2	-	-	-NO_2	-	-	-SO_2NHCH_3	-	33.41	5.53	0.0015
**32**	ChemDiv Inc.	8003-3805	-NO_2	-	-	-NO_2	-	-	-$SO_2N(CH_3)_2$	-	14.67	2.99	0.2570
**33**	ChemDiv Inc.	8004-6146	-NO_2	-	-	-NO_2	-	-	-$SO_2N(C_2H_5)_2$	-	41.56	5.92	0.0003
**34**	ChemDiv Inc.	8003-3814	-NO_2	-	-	-NO_2	-	-	-SO_2NHPh	-	6.21	3.80	0.9556
**35**	ChemDiv Inc.	8003-3793	-NO_2	-	-	-NO_2	-	-	-SO_2NH-3-$PhCH_3$	-	10.58	2.37	0.3013
**36**	ChemDiv Inc.	8003-3791	-NO_2	-	-	-NO_2	-	-	-SO_2NH-4-$PhCH_3$	-	-6.56	1.44	0.2919
**37**	ChemDiv Inc.	8003-3825	-NO_2	-	-	-NO_2	-	-	-SO_2NH-4-PhC_2H_5	-	9.96	5.55	0.9496
**38**	ChemDiv Inc.	8003-3787	-NO_2	-	-	-NO_2	-	-	-SO_2NH-4-$PhOCH_3$	-	12.29	5.99	0.7993
**39**	ChemDiv Inc.	8004-6133	-NO_2	-	-	-NO_2	-	-	-SO_2NH-4-PhCl	-	-5.82	5.05	0.9902
**40**	ChemDiv Inc.	8003-3799	-NO_2	-	-	-NO_2	-	-	-SO_2NH-4-PhBr	-	-23.90	9.63	0.6541
**41**	ChemDiv Inc.	8004-3396	-	-NO_2	-NO_2	-	-	-	-NO_2	-	10.26	3.18	0.2430
**42**	ChemDiv Inc.	8004-3394	-	-NO_2	-NO_2	-	-	-NO_2	-	-	-1.50	5.94	0.9999
**43**	ChemDiv Inc.	8004-3312	-	-NO_2	-NO_2	-	-	-CH_3	-	-	5.18	3.85	0.9685
**44**	ChemDiv Inc.	8004-3439	-	-NO_2	-NO_2	-	-	-CH_3	-	-CH_3	2.15	5.78	0.9997
**45**	ChemDiv Inc.	8004-3313	-	-NO_2	-NO_2	-	-CH_3	-	-	-CH_3	13.69	5.19	0.6311
**46**	ChemDiv Inc.	8004-3393	-	-NO_2	-NO_2	-	-CH_3	-	-NO_2	-	-2.17	5.67	0.9996

**47**	ChemDiv Inc.	8004-6017	-	$-NO_2$	$-NO_2$	-	-	$-CF_3$	-	-	-2.29	5.18	0.9998
**48**	ChemDiv Inc.	8004-3374	-	$-NO_2$	$-NO_2$	-	-	-	$-N(CH_3)_2$	-	8.70	0.88	0.0720
**49**	ChemDiv Inc.	8004-3329	-	$-NO_2$	$-NO_2$	-	-	-	-OH	-	10.06	2.34	0.3207
**50**	ChemDiv Inc.	8002-6832	-	$-NO_2$	$-NO_2$	-	-	-COOH	-OH	-	11.36	4.26	0.5330
**51**	ChemDiv Inc.	8004-3434	-	$-NO_2$	$-NO_2$	-	-	-	-COOH	-	10.79	2.26	0.2664
**52**	ChemDiv Inc.	8004-3444	-	$-NO_2$	$-NO_2$	-	-Cl	-	-	-	12.63	2.84	0.3036
**53**	ChemDiv Inc.	8004-3328	-	$-NO_2$	$-NO_2$	-	-I	-	-	-	8.95	3.28	0.5098
**54**	ChemDiv Inc.	0918-1055	-	$-NO_2$	-	-	-	-	$-NO_2$	-	23.70	2.28	0.0652
**55**	ChemDiv Inc.	8003-3783	-	-	-Br	$-NO_2$	-	$-NO_2$	-	-	10.58	1.75	0.0407
**56**	ChemDiv Inc.	2372-2057	-	$-NO_2$	-	-	-	-	$-S-4-PhNO_2$	-	16.32	16.32	0.0351

Table S3. BIP-4 analogs (group 3).

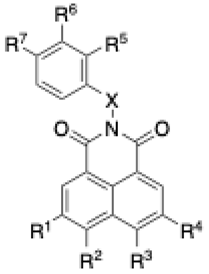

Compound number	Supplier	Supplier ID	R^1	R^2	R^3	R^4	X (N-X-C)	R^5	R^6	R^7	% inhibition[1]	SD	P value
**57**	ChemDiv Inc.	8004-3423	$-NO_2$	-	-	$-NO_2$	$-CH_2-$	-	-	-	11.32	2.11	0.2207
**58**	ChemDiv Inc.	8010-1858	-	-	-	-	-CO-	$-NO_2$	-	$-NO_2$	11.61	1.39	0.0987
**59**	ChemDiv Inc.	8001-6847	-	$-NO_2$	-	-	-NH-	$-NO_2$	-	$-NO_2$	6.66	5.81	0.9987
**60**	ChemDiv Inc.	8004-3379	-	$-NO_2$	$-NO_2$	-	-NH-	$-CH_3$	-	-	38.02	6.86	0.0025
**61**	ChemDiv Inc.	8004-3376	-	$-NO_2$	$-NO_2$	-	-NH-	-	$-CH_3$	-	1.00	3.78	0.9999
**62**	ChemDiv Inc.	8004-3381	-	$-NO_2$	$-NO_2$	-	-NH-	-	-	$-CH_3$	16.42	2.19	0.1205
**63**	ChemDiv Inc.	8004-3389	-	$-NO_2$	$-NO_2$	-	-NH-	-	$-CH_3$	$-CH_3$	18.57	3.18	0.1899
**64**	ChemDiv Inc.	8004-3386	-	$-NO_2$	$-NO_2$	-	-NH-	$-CH_3$	-	$-CH_3$	19.89	2.57	0.1135
**65**	ChemDiv Inc.	8004-3347	-	$-NO_2$	$-NO_2$	-	-NH-	-	-Cl	-	23.80	5.26	0.2953
**66**	ChemDiv Inc.	8001-6848	-	$-NO_2$	-	-	-NHCO-	$-NO_2$	-	-	9.99	7.58	0.9723
**67**	ChemDiv Inc.	8004-6000	-	$-NO_2$	$-NO_2$	-	-NHCO-	$-NO_2$	-	-	-5.65	4.24	0.9705
**68**	ChemDiv Inc.	8004-3340	-	$-NO_2$	$-NO_2$	-	-NHCO-	-	-	$-NO_2$	-15.08	23.29	0.9997

**69**	ChemDiv Inc.	8004-3367	-	$-NO_2$	$-NO_2$	-	-NHCO-	-	-	-OH	-5.02	2.51	0.8141
**70**	ChemDiv Inc.	8003-3722	$-NO_2$	-Br	-	-	-NHCO-	-	$-NO_2$	-	-31.54	11.83	0.5332
**71**	ChemDiv Inc.	8004-3358	$-NO_2$	-	-	$-NO_2$	$-NHCOCH_2-$		-	-	10.72	5.25	0.8017
**72**	ChemDiv Inc.	0645-0170	-	$-NO_2$	-	-	-N=C-	-	$-NO_2$	-	6.16	.94	0.9947
**73**	ChemDiv Inc.	1189-2520	-	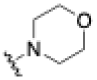	-	-	$-C_3H_6OCO-$	$-NO_2$	-	$-NO_2$	12.58	2.47	0.2416
**74**	ChemDiv Inc.	8004-3441	-	-	-	-	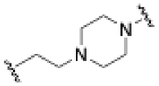	$-NO_2$	-	$-NO_2$	-16.99	3.24	0.2305

Table S4. BIP-4 analogs (singletons).

Compound number	Supplier	Supplier ID	Structure	% inhibition[1]	SD
**75**	ChemBridge Corp.	STL397142	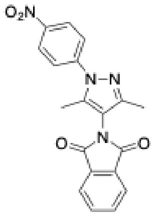	10.35	2.63
**76**	ChemDiv Inc.	8004-3441	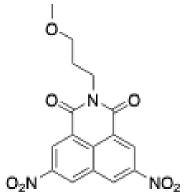	-1.76	3.86
**77**	ChemDiv Inc.	8008-4145	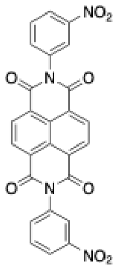	-6.79	4.49
**78**	ChemDiv Inc.	8004-3450	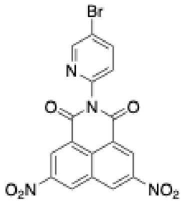	1.60	4.61
**79**	ChemDiv Inc.	8005-0121	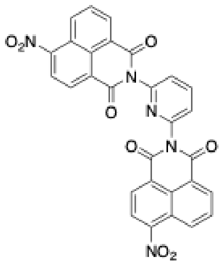	-16.67	9.10
**80**	ChemDiv Inc.	0547-0180	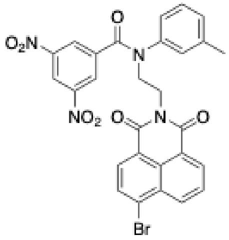	16.82	3.89

Table S5. Inhibition activity and statistical significance of active compounds.

	Compound number	Supplier	Supplier ID	Structure	% inhibition[2]	SD	P value	n
**group 1**	**BIP-4**	ChemDiv Inc.	8010-1372	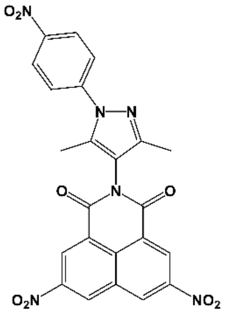	18.94	1.48	<0.0001	4
	**5**	ChemBridge Corp.	7045306	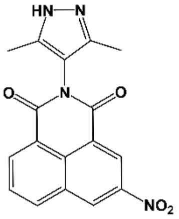	18.21	1.81	<0.0001	8
	**7**	ChemBridge Corp.	7093221	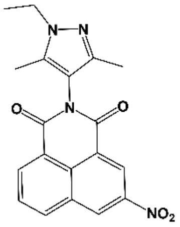	16.22	3.12	0.0313	3
	**9**	ChemBridge Corp.	7053478	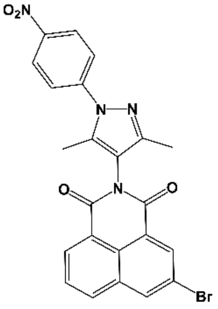	15.53	2.20	<0.0001	8

[2] With 5µM Compound, measured with ADP Glo™ Assay, normalized to positive control

	**14**	ChemDiv Inc.	8010-2470	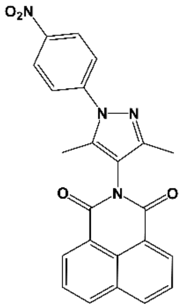	12.13	2.33	0.0310	3
**group 2**	**19**	ChemDiv Inc.	8003-3839	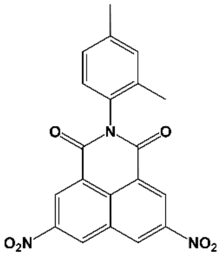	36.22	4.04	<0.0001	7
	**27**	ChemDiv Inc.	8003-3844	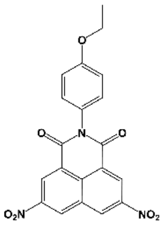	31.43	3.34	<0.0001	7
	**28**	ChemDiv Inc.	8004-3310	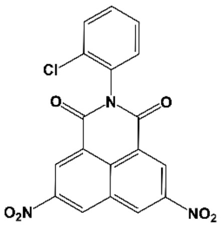	19.66	3.34	0.0006	6
	**31**	ChemDiv Inc.	8003-3811	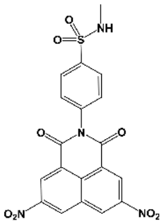	33.41	5.53	0.0015	6
	**33**	ChemDiv Inc.	8004-6146	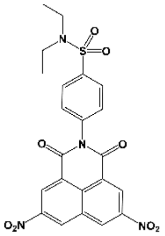	41.56	5.92	0.0003	6

	**55**	ChemDiv Inc.	8003-3783	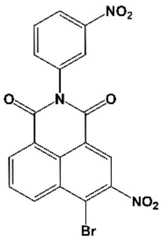	10.58	1.75	0.0407	3
	**56**	ChemDiv Inc.	2372-2057	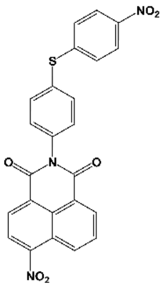	16.32	16.32	0.0351	3
**group 3**	**60**	ChemDiv Inc.	8004-3379	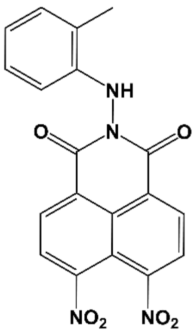	38.02	6.86	0.0025	6
